# 

**Published:** 1889-12

**Authors:** 


THE MEDICAL NEWS & VISITING LIST, 1890. Thirty patients per week. Lea Brothers & Co.

This is an admirable visiting list for the physician. Revised and improved from former editions. It contains much useful memoranda and information, with table of doses. Therapeutic table calendar; ligation of arteries, incompatibles; obstetric table; weights and measures; catheter scale, etc., etc.

